# Gestational Diabetes Mellitus Risk Optimisation in Pregnant Women Prescribed Atypical Antipsychotics: Insights from a Three-Cycle Plan-Do-Study-Act Quality Improvement Project Across South East London

**DOI:** 10.1192/j.eurpsy.2026.11922

**Published:** 2026-07-31

**Authors:** S. Uthayakumar, S. Lewis, A. Qureshi, J. Yasmin, J. Krens, A. Delvi

**Affiliations:** 1 Faculty of Life Sciences and Medicine; 2 King’s College London; 3 Oxleas NHS Foundation Trust, London, United Kingdom

## Abstract

**Introduction:**

South East London (Oxleas) Perinatal Mental Health Services (PMHS) recommend an Oral Glucose Tolerance Test (OGTT) at 14-16 weeks, in addition to the National Institute for Health and Care Excellence (NICE) recommended 28-week OGTT for pregnant women prescribed atypical antipsychotics. Oxleas’ early screening pathway, developed in collaboration with King’s College Hospital NHS Foundation Trust, aims to facilitate earlier identification and management of Gestational Diabetes Mellitus (GDM).

**Objectives:**

This project aims to evaluate current GDM optimisation practices amongst responsible clinicians and midwives, following Standards for Quality Improvement Reporting Excellence guidelines.

**Methods:**

To evaluate baseline documentation practices, a retrospective database audit was conducted on RiO, the Trust’s electronic patient record system, from November to December 2024. Three low-resource, education-focused Plan-Do-Study-Act Cycles followed. Cycle One involved a student-led educational session, followed by questionnaires assessing perinatal psychiatrists’ recall of GDM screening principles i.e., indication, fasting, and NICE vs Trust OGTT timings. Cycle Two involved an infographic prompt (Image 1), distributed to all responsible midwives, with semi-structured interviews assessing effectiveness. Following feedback, the infographic was displayed at all three Oxleas sites and shared with clinicians. Cycle Three revised the Trust’s assessment template to prompt documentation of OGTT booking and results on RiO, at both 14-16 and 28 weeks. Effectiveness will be evaluated in January 2026, by comparing documentation rates in a new cohort of pregnant women on atypical antipsychotics against baseline, accounting for sample size variation.

**Results:**

Of the 47 pregnant women on atypical antipsychotics, 34% (n=16) had a documented 28-week OGTT, result, and management plan. No patient had the Trust-recommended 14-16-week OGTT. After Cycle One, all clinicians (n=5) were able to recall GDM screening principles and 28-week OGTT, but only one clinician recalled the 14-16-week OGTT. Midwife absences limited Cycle Two data, however, two respondents reported that the infographic positively influenced their practice due to prior unfamiliarity with antipsychotic risks and GDM screening.

**Image 1:**
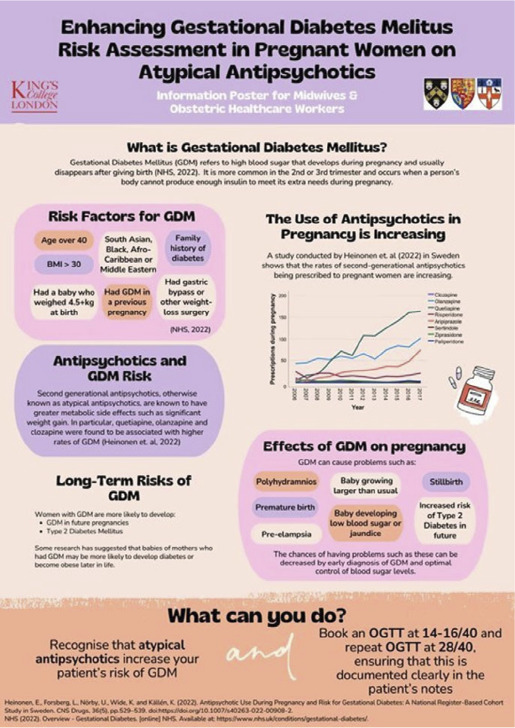
Image 1: Long description.

**Conclusions:**

Limited clinician and midwife awareness of the Trust’s OGTT guidance, plus discrepancies between national and Trust recommendations may contribute to suboptimal identification, management and documentation of GDM risk in pregnant women prescribed atypical antipsychotics across Oxleas. Integrating clinician- and midwife-targeted education, infographic prompts and Trust-wide assessment template amendments, offer a scalable strategy adaptable to national and international PMHS’ provision, to optimise GDM risk in this group.

**Disclosure of Interest:**

None Declared

## Long descriptions

### Image 1: Long description

The poster is titled Enhancing Gestational Diabetes Mellitus Risk Assessment in Pregnant Women on Atypical Antipsychotics.

Top Section:

- Header includes King's College London logo and three crests.

- Definition: G D M refers to high blood sugar during pregnancy, common in the 2nd or 3rd trimester.

Middle Left Column:

- Risk Factors for G D M: Age over 40, B M I greater than 30, South Asian, Black, Afro-Caribbean or Middle Eastern ethnicity, family history of diabetes, previous baby weighing 4.5 plus kg, previous G D M, or gastric bypass surgery.

- Antipsychotics and G D M Risk: Second-generation antipsychotics like quetiapine, olanzapine, and clozapine are linked to significant weight gain and higher G D M rates.

- Long-Term Risks: Increased likelihood of G D M in future pregnancies and Type 2 Diabetes Mellitus for the mother, and potential obesity for the child.

Middle Right Column:

- Line Graph: Shows prescriptions during pregnancy from 2006 to 2017. Quetiapine shows a sharp linear increase from approximately 50 to 175 prescriptions. Other drugs like Olanzapine and Risperidone show gradual increases or stable low rates.

- Effects of G D M on pregnancy: Lists Polyhydramnios, Premature birth, Pre-eclampsia, Baby growing larger than usual, Baby developing low blood sugar or jaundice, Stillbirth, and increased risk of Type 2 Diabetes in the future.

Bottom Section:

- Call to Action: Recognise that atypical antipsychotics increase risk. Book an O G T T at 14 to 16 over 40 and repeat O G T T at 28 over 40.

- References: Cites Heinonen et al. 2022 and N H S 2022.

Navigate back to Image 1:.

